# Non-Fatal Attacks by Dogs: Characteristics of Victims and Attacking Dogs, From the Forensic Perspective: A Series of 106 Cases From Athens, Greece, and Brief Review of the Literature

**DOI:** 10.7759/cureus.21097

**Published:** 2022-01-10

**Authors:** Dimitrios Kouzos, Konstantinos Katsos, Evmorfili I Zouzia, Konstantinos Moraitis, Dimitrios G Vlachodimitropoulos, Nikos Goutas, Chara A Spiliopoulou, Emmanouil I Sakelliadis

**Affiliations:** 1 Forensic Medicine and Toxicology, National and Kapodistrian University of Athens, School of Medicine, Athens, GRC; 2 First Intensive Care Clinic, National and Kapodistrian University of Athens, School of Medicine, Athens, GRC

**Keywords:** greece, injury assessment, dog bites, animal attacks, forensic clinical examination

## Abstract

Introduction: The close association of dogs with humans may explain the fact that dog bites are possibly the most common animal bites recorded. The relevant data concerning Greece is scarce. We aimed to study this phenomenon by describing its characteristics.

Methods: We performed a retrospective analysis of cases concerning dog attack victims, examined our Department, between 2011 and 2019. Recorded variables included sex, age, nationality, occupation, marital status, medical history, ownership status of the dog, injury anatomic location, incident time, the timing of clinical forensic examination, incapacitation time, and medical care provided.

Statistical analysis was performed using Stata/MP 13 (Stata Corp., College Station, TX) and IBM SPSS Statistics Version 20 (IBM, Armonk, NY). Statistical significance was defined as a two-sided p value of <0.05.

Results: Most incidents involved male victims (54.2%). The victim’s mean age was 44.9 years. The dog involved was unowned in 19.8% of cases. The most frequent anatomical site of injury was the legs (48.1%). Older victims suffered injuries in more sensitive areas of the body (head and neck), when compared to younger adults. Only 1.9% of victims required hospitalization. The mean incapacitation time was estimated at 5.39 days.

Conclusions: Per our results, males tend more often to be victims of dog attacks. Typically, victims are of increased age and are attacked by a dog already known to them. Most incidents take place during late winter and spring, more specifically during February and during May. The most frequently affected anatomical sites were the legs. Older people suffered injuries in more sensitive areas of the body.

## Introduction

Bitemarks are generally defined as a pattern, produced by teeth in a substrate. Animal bites may exhibit wide variation depending on various factors (animal involved, attack circumstances, and victim’s characteristics) [1]. Injuries inflicted by dogs have a reported prevalence of 0.3%-1.1% of all emergency department (ED) visits and in some cases, they may cause death [2].

There is an ongoing debate about which dogs attack humans the most. Some conclude that high-risk animals include larger dogs such as German shepherds, pit bulls, and Rottweilers; but nevertheless, all dogs may inflict severe injuries [3]. On the other hand, no significant difference in biting frequency exists, between pedigree and mixed-breed dogs. Male dogs account for approximately 75% of all incidents and behave more aggressively [4]. According to the literature, freely roaming dogs represent a community health risk [5].

It appears that many attacks are unprovoked, but some incidents may be linked to disturbance of the dog during eating, to threats, to the invasion of its territory, or even to jealousy of attention given to other family members [3, 6].

Approximately half of the children population will sustain a dog bite sometime during their lives, most often to the face, neck, and head [1, 7]. On the other hand, in incidents involving adults, only 10% of bites are located on the head and neck [3]. Injuries to the extremities are described to account for most dog-related injuries [2].

It has been reported that the most common victims of dog bites are males and children or adolescents [4]. In Spain, children in the age group 0-14 were at risk three times more than people aged 15 years or more [8]. Dogs known to the victim seem to be more frequently involved in incidents [6, 8].

Extensive litigation may follow a dog bite incident, either against the dog owner or even against the physician who provided medical attention [3]. In Greece, a forensic clinical examination is mandatory in every allegation of bodily harm, to assess injury severity, according to the Greek Penal Code (GPC). Furthermore, every person who causes bodily harm by negligence (e.g., an owner of a dog) can be punished by offering social work, by paying a fine, or by imprisonment for up to two years.

The aim of our study is to identify characteristics, associated with dog-bite incidents, either concerning the victim or the attacking dog.

## Materials and methods

Victims of dog attacks that underwent forensic clinical examination, during the period 2011-2019, performed at the Department of Forensic Medicine and Toxicology of the Medical School at the National and Kapodistrian University of Athens were included in our study sample.

The recorded variables included sex, nationality, occupation, marital status, possible diseases that the victims suffered from, and age. Regarding age, as minors, we defined those who were younger than 18 years old (group A). The bulk of adults was defined as 18-67 years old (group B), while older adults were defined as being older than 67 years (group C) (this age matches the mandatory retirement age limit in Greece).

Psychiatric disorders included depression, anxiety disorders, psychosis, dementia, and bipolar disease. Neurologic disorders included hearing impairment and hydrocephalus. Mobility disorders included all musculoskeletal diseases that resulted in reduced mobility.

Additionally, the ownership status of the attacking dog (unowned or owned) was recorded, as well as the anatomic injury location. Furthermore, the time of the incident was included in the analysis, as well as details concerning the timing of the clinical forensic examination, the time period of incapacitation due to injuries, as estimated by the forensic pathologist, and finally the means of medical care, if any, that the victim received.

Statistical analysis was performed using Stata/MP 13 (Stata Corp., College Station, TX) and IBM SPSS Statistics Version 20 (IBM, Armonk, NY). Statistical significance was defined as a two-sided p value of <0.05.

Descriptive results (e.g., frequency) were reported for each variable. Categorical variables were reported as numbers and percentages. Continuous Gaussian variables were reported as means with standard deviation (SD) and non-Gaussian variables as medians with interquartile range (IQR). Pearson chi-square test and t-test (for categorical and continuous variables respectively) were appropriately used when comparison among variables was performed. Due to the relatively small size of our sample whenever Pearson’s chi-square test was not applicable Fischer’s exact test was used.

A hypothesis of a potential seasonal variation of dog attacks was made. A chi square goodness-of-fit test was applied to examine whether there was heterogeneity in the number of dog attacks among specified time intervals, testing thus the occurrence of any significant departure from a uniform distribution. A second analysis was performed in order to detect the presence of seasonality, in this case, Edward’s seasonality test, a statistical method specifically designed to detect seasonality and cyclic trends [9]. In order to achieve this, given the limited sample size, time was categorized in the following intervals: four-hour intervals (e.g., 00:00-04:00), days of weeks (Monday-Sunday), months, and seasons.

## Results

Demographic characteristics

Male predominance of victims (54.2%) was a noteworthy characteristic. The mean age of victims was 44.9 years. Additionally, most victims (43.4%) were married. White collar workers (29.2%) or retired (28.3%) represented the two most common victims’ occupations. For detailed results, please refer to Table 1. 

**Table 1:** Demographic characteristics of dog-bite incident victims. SD, standard deviation;

Parameter examined	Results
Age (mean/median) ± SD years	(44.89/46) ± 19.759
Age range (min/max)	80 (4/84)
Age groups	Minors (age <18)= 11 (10.4%)
	Young adults (age 18-67)= 79 (74.5%)
	Older adults (age >67)= 16 (15.1%)
Gender	Male= 58 (54.2%)
	Female= 48 (45.3%)
Nationality	Greek= 98 (92.5%)
	Other= 8 (7.5%)
Marital status	Single= 35 (33%)
	Married= 46 (43.4%)
	Divorced= 9 (8.5%)
	Widowed= 4 (3.8%)
	Unknown= 12 (11.3%)
Occupation	Blue collar= 14 (13.2%)
	White collar= 31 (29.2%)
	Unemployed= 7 (6.6%)
	Retired= 30 (28.3%)
	Student= 19 (17.9%)
	Unknown= 5 (4.7%)

Analysis specially performed for the detection of a pre-existing disease yielded the following findings: arterial hypertension (12.3%), coronary artery disease (9.4%), psychiatric neurologic disorder (6.6%), diabetes mellitus, and mobility issues (3.8% each). All relevant data were obtained from the medical history provided by each victim. Further analysis revealed, as it was expected, a significant difference age-wise; as 20% of group B vs. 69% of group C apparently had at least one pre-existing disease (p < 0.001, Fisher's exact test).

The dog involved in the incident was owned in 49.1% of the cases examined, while in 19.8% the dog was unowned. Unfortunately, in 31.1% of the cases, the victim was not able to provide further information as to the dog.

Regarding the status of the dog, it seems that a non-definite tendency of group C to be attacked more often than group B by unowned dogs exists (60% vs. 24.5% respectively) (p < 0.054, Fisher's exact test).

There were no significant differences between male and female victims regarding the ownership status of the dog (unowned or owned) [X2 (1, N=73) = 0.033 p=0.856)].

**Figure 1:**
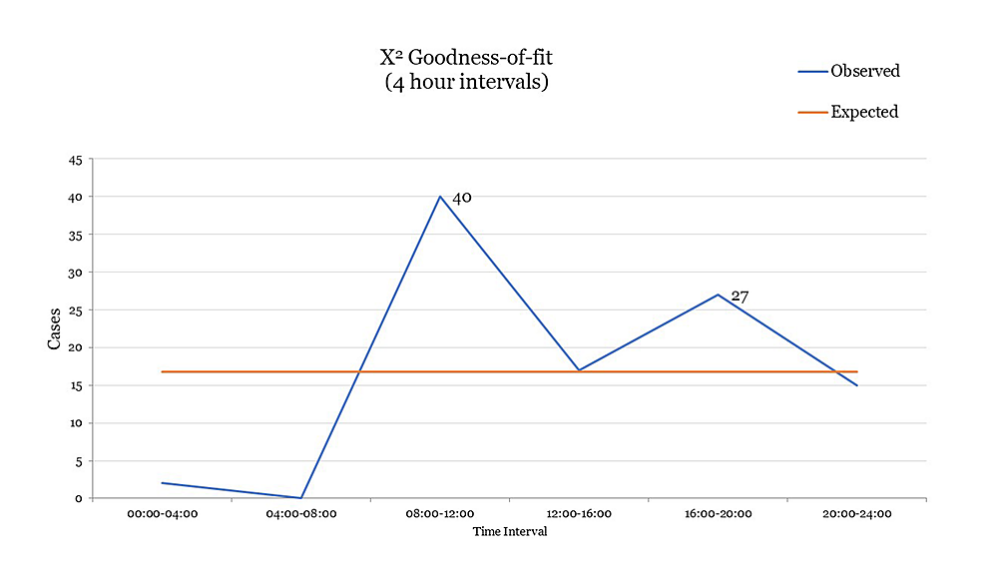
Time of the day. Time of the day when dog bite attacks from the examined cases occurred. X2=68.63, df=5, p=0.001.

Regarding the distribution of cases through the day, there was heterogeneity in the frequency of events with the peak between 08:00 and 12:00, a second lower peak between 16:00 and 20:00, and the through between 00:00 and 08:00 (X2=68.63, df=5, p=0.001) (Figure 1).

There was no significant variability in the weekly distribution (X2=7.32, p=0.292). 

**Figure 2:**
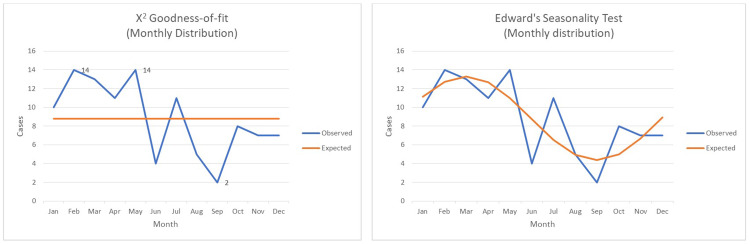
Monthly distribution and seasonal trend. Monthly distribution and seasonal trend of dog-bite incidents. To the left a chi-square goodness-of-fit test revealed the presence of heterogenicity in the monthly distribution of cases. To the right Edwards’ test revealed a cyclic trend of the same parameter.

As to the monthly distribution of dog-bite incidents, there was heterogeneity in the frequency of cases (X2=19.66, df=11 p=0.05) with two observed peaks in February and May and a through in September. Edward’s test was performed to detect the presence of seasonality. The monthly distribution of cases appears to follow a simple harmonic curve pattern that reaches a peak in March and a through in September. Specifically, the amplitude of the expected frequency curve was 0.51. No significant departure of observed frequencies from the fitted simple harmonic curve was detected (X2=10.43, p=0.492) (Figure 2).

Concerning seasonal distribution, heterogeneity was also detected performing chi-square goodness-of-fit method with the peak during Spring and the through during Autumn (X2=10.75, df=3 p=0.01). The incident site, according to the victim, was by majority a public road or a pavement (64.2%) (Table 2).

**Table 2:** Incident site distribution. *Public places consist of squares, parks, a beach, a sports field, a super-market parking, a cemetery, and a restaurant.

	N (%)
Public road/pavement	68 (64.2)
Other public spaces*	15 (14.2)
Private spaces	9 (8.5)
Rural areas	3 (2.8)
Unknown	11 (10.4)

Anatomical distribution of injuries

The most frequent anatomical sites of injuries were the legs (48.1% of the cases), the thighs (36% of the cases) and the hands (20.8% of the cases), as described in detail in Table 3.

**Table 3:** Anatomic distribution of injuries.

	N (%)
Head	2 (1.9)
Face	3 (2.8)
Neck	2 (1.9)
Torso (anterior surface-thorax)	2 (1.9)
Torso (abdomen)	4 (3.8)
Torso (posterior surface)	5 (4.7)
Genitals	1 (0.9)
Arms	10 (9.4)
Forearms	17 (16)
Hands	22 (20.8)
Thighs	36 (34.0)
Legs	51 (48.1)
Feet	10 (9.4)

The site of injury was also evaluated, taking under consideration the status of the attacking dog (unowned or owned). Upper extremity injuries were more frequent in victims of owned dogs' attacks [X² (1, N=73) = 5.850, p=0.016] while lower extremity injuries were more common among those that were attacked by unowned dogs [X² (1, N=73) = 7.594, p=0.008].

Regarding the anatomic location affected, group C suffered bites in more sensitive areas of the body (head and neck), in comparison to group B (Fisher’s exact test p=0.014 and p=0.027 respectively). Regarding injury distribution on the torso, the upper and the lower extremities, no statistically significant difference was observed.

Finally, no injuries were recorded on the head, face, or neck of group A that were examined.

Treatment

Most victims sought medical treatment after the dog-bite incident as shown in Table 4.

**Table 4:** Treatment types offered by medical personnel after a dog bite.

	N (%)
First aid	Yes= 67 (63.2)
	No= 37 (34.9)
	Unknown= 2 (1.9)
Hospitalization	Yes= 2 (1.9)
	No= 102 (96.2)
	Unknown= 2 (1.9)
Suturing	Yes= 16 (15.1)
	No= 90 (84.9)
Antibiotics	Yes= 52 (49.1)
	No= 47 (44.3)
	Unknown= 7 (6.6)
Anti-tetanic serum	Yes= 41 (38.7)
	No= 58 (54.7)
	Unknown= 7 (6.6)
Medical facility	None= 34 (32.1)
	Private facility= 11 (10.4)
	Public facility= 51 (48.1)
	Unknown= 10 (9.4)
Types of medical facility	None= 34 (32.1)
	Private practice= 4 (3.8)
	Health centre= 12 (11.3)
	Hospital= 46 (43.4)
	Unknown= 10 (9.4)

However, only 1.9% of them required hospitalization. Public facilities, more specifically hospitals, were preferred more often by the victims. A hypothesis that victims may choose a different kind of facility depending on their age was made, but our analysis led to its rejection. A second hypothesis that the required treatment is age-dependent was made. However, it was also rejected as no statistically significant differences were observed.

The mean estimated incapacitation period was 5.39 days. A forensic examination was performed at a mean time of 2.64 days after the incident.

An independent-sample t-test was conducted to compare the estimated incapacitation period between male and female victims. There was not any significant difference in the scores for females (M=6.49, SD=6.814) and males (M=4.46, SD=4.085) [t (101) = -1.862, p=0.066].

An independent-sample t-test was conducted to compare the estimated incapacitation period in group B and group C. There was not any significant difference in the scores for younger (M=5.26, SD=5.342) and older adults (M=7.2, SD=7.8) [t (90) = -1.187, p=0.239].

There were not any significant differences between those attacked by unowned vs. owned dogs, regarding the pursuit of medical care [X² (1, N = 66) = 0.520, p < 0.471].

Furthermore, a comparison regarding attacks of unowned vs. owned dogs on minors vs. adult victims was made, but no statistically significant difference was detected (Fisher’s exact test p-value = 0.666).

The total number of injuries inflicted on each victim caused by unowned vs. owned dogs was also compared without, however, the detection of any statistically significant difference between the two groups (Fisher’s exact test p-value = 1).

## Discussion

Various parameters of dog-bite incidents were examined to identify the specific characteristics, either concerning the victim or the attacking dog, and to compare them with current available literature.

Victim characteristics (gender, age, occupation, and previous medical history)

Male victim predominance, reported by other studies is verified by our results as well [8, 10-11].

Although many studies suggest that minors present increased incidence, our results demonstrate the opposite [3, 10, 12-13]. More specifically, in our sample, only 10.4% of cases pertained to minors.

While the low socio-economic status of the victim is well described in the literature, white-collar workers (29.2%) and retired individuals (28.3%) constitute the main social groups that requested forensic clinical examination in our department [8, 11-12]. We believe that low socio-economic status victims do not easily engage in litigation, especially for minor issues such as a dog bite.

Although people suffering from mental illness were previously reported to be more prone to dog attacks, mainly because their behavior can often be misunderstood by a dog as aggressive, only 6.6% of victims in our sample reported a history of mental illness [8, 13].

Attacking dog characteristics

As to the dog ownership status, our results indicate that in 49.1% of cases the attacking dog was owned, while in 19.8% it was unowned. Lack of data for the remaining cases prohibited their inclusion in either subgroup regarding this matter. Furthermore, available data did not include dog race, so no analysis concerning its impact on incidents was feasible.

Incident characteristics (location and timing)

Literature establishes well that incident location is more often in public spaces, a finding that is consistent with our analysis, which demonstrated that most incidents took place either on a public road or pavement [8, 14].

Concerning time intervals of dog attacks, our analysis demonstrated two frequency peaks throughout the day (08:00-12:00 and 16:00-20:00). This can possibly be explained by the owners’ habit to walk their dogs during these time intervals, thus increasing the number of dogs in the streets and consequently the possibility of human-dog interaction.

Our analysis determined that dog-bite incidents take place more often during late Winter-early Spring, a finding that is not consistent with a large retrospective study from the United States, which describes a peak during summer [10]. This is possibly explained by the fact that in Greece during late winter and springtime, the climate is milder, compared to other countries, again facilitating human-dog interaction. Moreover, the low incidence during summertime in our study may be explained by the fact that many inhabitants in the Attica region are away on vacations.

Injuries inflicted

Current literature suggests that older individuals may sustain more often dog bites in their hands, during their effort to repel an attacking dog, while younger ones may find themselves trying to separate a dog fight or may interact roughly with them, in a way that may be perceived as aggressive by the animal, thus making themselves vulnerable to dog bites in their lower extremities [15]. However, the conditions which characterize each dog bite incident vary significantly, which possibly explains why injuries have been recorded in almost every anatomic location of the human body [16-17].

The results of our study did not demonstrate that minors sustain injuries more often on the head and neck, as described in the literature, perhaps because minors are under-represented in our sample (10.4% of the total sample) or because it is possible that these injuries may have been inflicted by a familiar owned dog, thus were not reported to the authorities [2, 10, 18].

The anatomic distribution of dog bite injuries in our sample (leg, thighs, hands) is in accordance with recent literature [8, 10, 12]. Nevertheless, differences exist, depending on the ownership status of the attacking dog. Owned dogs inflicted injuries more frequently on the upper extremities (possibly because victims had some degree of confidence when approaching the animal), while on the other hand, unowned dogs inflicted injuries more often on the lower extremities.

Regarding the distribution of injuries to more sensitive areas of the body (head and neck) in different age groups, our analysis indicated that elderly victims indeed sustain injuries in these areas, more often when compared to younger adults. This can probably be explained by loss of balance and consequently fall to the ground, in their attempt to avoid the attacking dog, thus granting access to these sensitive areas. Furthermore, in contrast to information derived from current literature, no injuries were recorded on the head, face, or neck of minors that were examined.

Medical care required

Literature suggests that dog-bite victims often seek medical treatment according to the severity of their condition. Approximately, 1.1% of ED admissions concern dog bite victims, but only a small proportion (1.7% of these cases) require further hospitalization [10]. A more thorough approach is often adopted in children, as a higher percentage of them visits an ED after a dog bite and consequently, hospital admissions occur more often too [12]. According to our analysis, only 63.2% of victims received first aid from medical personnel.

Dog-bite injuries can cause fractures, infections, vascular injuries, post-traumatic stress disorder (PTSD), or even death [3, 19-22]. After first aid, a dog-bite wound requires irrigation and debridement of the affected tissues [23].

The safety of primary wound closure in dog-bite victims is still under debate by surgeons. Recent literature suggests suturing a wound on the face but leaving open wounds in extremities to minimize the infection risk [3, 24]. Infections following such injuries may include rabies, infective endocarditis, and severe septicemia [25-26]. Especially for rabies post-exposure prophylaxis is almost 100% effective [26-27].

In general, the literature suggests that even minor infections can have devastating complications that may actually threaten the survival of the patient. Antibiotic prophylaxis is indicated in high-risk groups. These groups include individuals with primary wound closure, with edema, with crush injury, with excessive devitalized tissue, or with full-thickness wounds involving tendons, ligaments, and joints. Furthermore, those with puncture wounds, facial bites, hand or foot bites, genital area bites, and finally those who are immunocompromised or have undergone splenectomy are also included in high-risk groups after a dog bite [28]. Finally, tetanus immunization is advised in some cases following a dog bite [29].

Per our results 15.1% underwent primary wound closure, 49.1% were administered antibiotic prophylaxis, and finally, 38.7% had anti-tetanus immunization. No report of anti-rabies serum immunization was recorded, even though rabies cases are occasionally reported in the northern part of Greece [30]. It appears that therapeutic strategies employed by the medical personnel, in cases of our study, are in line with current literature.

Legal aspects

In the UK, dogs that inflict fatal injuries are often euthanized or sheltered after these incidents against the will of their owner and often even against the will of the victim of the dog bite as well [11]. In France, these dogs are set under the observation of a veterinary doctor who decides on their aggressiveness and then their fate is decided by administrative authorities [30]. Owner prosecution and compensation claims are also quite often a matter of litigation, especially in the cases when forensic medicine intervenes. In Greece, dog owners are liable for any damage inflicted by the dog. It becomes clear, therefore, that dog ownership requires a vast amount of responsibility towards oneself, others, and the dog itself.

In Greece, clinical forensic examination works as an independent and unbiased tool for the estimation of incapacitation time which results from incidents that cause bodily harm (e.g., dog bite). The mean estimated incapacitation period, was 5.39 days, while the mean timing of the forensic clinical examination was 2.64 days after the incident.

Limitations

The main limitation of our study is that as our jurisdiction area covers approximately half of the Athens Metropolitan Area, mainly an urban area, incidents occurring in rural areas might be under-reported in our sample. Furthermore, cases examined in our department, include only those victims that reported the incident to the Police, thus it is expected that the actual incident number is much greater than the one included in our sample, for the time period examined.

## Conclusions

Per our results, males tend more often to be victims of dog attacks, when compared to females. Typically, victims are of increased age and are attacked by a dog already known to them. Regarding seasonal distribution, most incidents take place during late winter and spring. The monthly distribution of cases demonstrated that most incidents took place during February and during May. Weekly distribution did not present any statistically significant variability. As to the case distribution throughout the day, we detected two peaks, the first between 08:00 and 12:00 and the second lower peak between 16:00 and 20:00. In our sample, most incidents took place in a public space. The most frequently affected anatomical sites were the legs. Older people suffered injuries in more sensitive areas of the body. Forensic clinical examination was performed at a mean time of 2.64 days after the incident. Finally mean incapacitation period, after a dog-bite incident was estimated in 5.39 days.
